# Supplementary material for: Genome sequencing unveils blaKPC-2-harboring plasmids as drivers of enhanced resistance and virulence in nosocomial Klebsiella pneumoniae
Source: mSystems. 2024 Jan 9;9(2):e00924-23. doi: 10.1128/msystems.00924-23 (PMC10878039; doi:10.1128/msystems.00924-23)
Supplement: Supplemental figures — Fig. S1 and S2. [file msystems.00924-23-s0001.pdf]

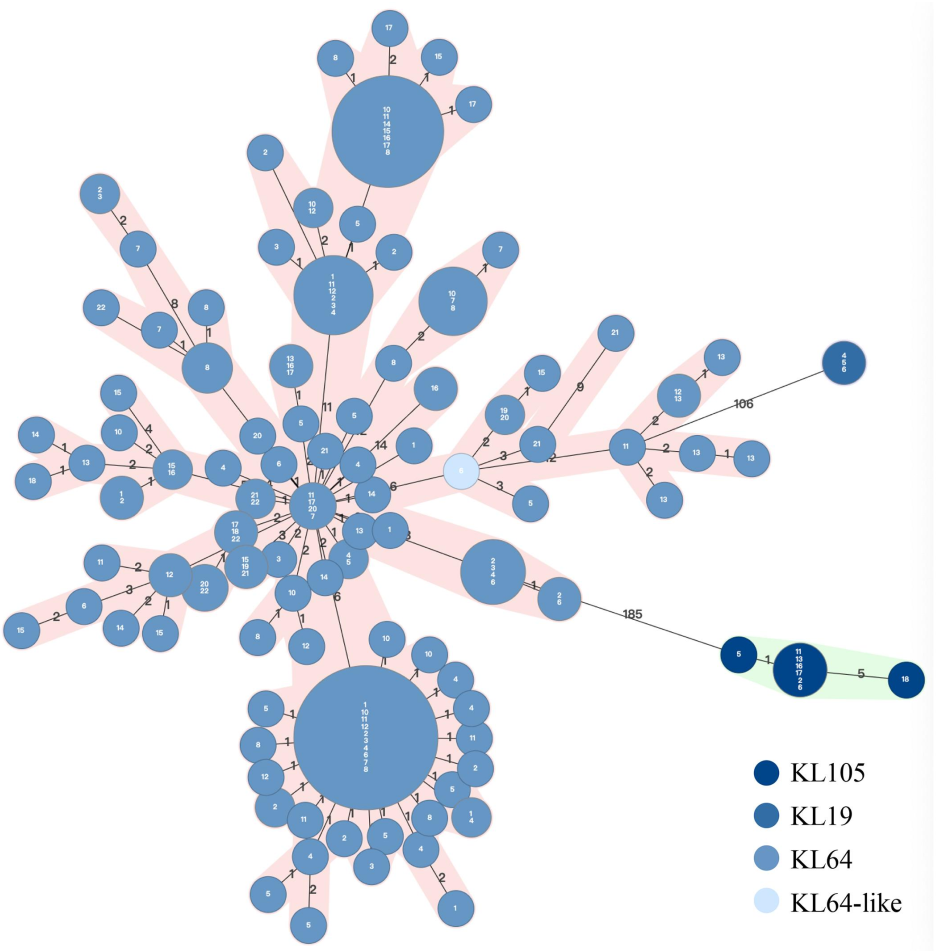

Fig. S1. Minimum spanning tree of 237 ST11-KPC-2-CRKP isolates. Generated by Ridom SeqSphere+ software version 7.2.3 (Ridom GmbH, Muenster, Germany) using the cgMLST scheme. The isolates were divided into three clusters, painted with different colors in the background. Nodes were painted according to serotype.

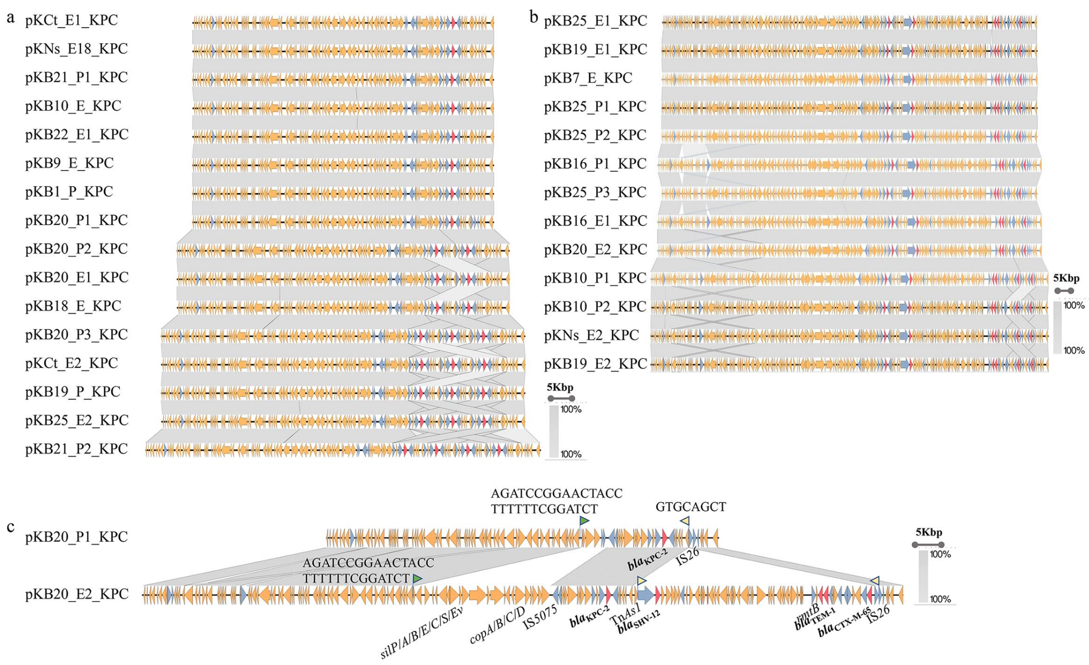

Fig. S2. Linear structure of IncFII(pHN7A8)/IncR type pKPC-2 plasmids. a, Comparison and structure of subgroup 1 IncFII(pHN7A8)/IncR-type pKPC-2; **b,** Comparison and structure of subgroup 2 IncFII(pHN7A8)/IncR-type pKPC-2; **c,** Linear comparison of subgroups 1 and 2 pKPC-2. Red arrows indicate ARGs, blue arrows indicate IS elements or Tn transposons and yellow arrows indicate CDSs.
