# Supplementary material for: Genome sequencing unveils blaKPC-2-harboring plasmids as drivers of enhanced resistance and virulence in nosocomial Klebsiella pneumoniae
Source: mSystems. 2024 Jan 9;9(2):e00924-23. doi: 10.1128/msystems.00924-23 (PMC10878039; doi:10.1128/msystems.00924-23)
Supplement: Supplemental tables — Tables S1 and S2. [file msystems.00924-23-s0002.docx]

Table S1 Estimated copy number and MIC of novel β-lactamase/β-lactamase inhibitor combinations in 237 ST11 KPC-CRKP

| ID | ST | KPC-2 | Estimated KPC-2 copy number | SHV-12 | Estimated SHV-12 copy number | CTX-M-65 | Estimated CTX-M-65 copy number | CZA | IMR | MEV |
| --- | --- | --- | --- | --- | --- | --- | --- | --- | --- | --- |
| kp1 | 11 | + | 1.84 | nd | nd | nd | nd | 2 | 0.25 | 1 |
| kp2 | 11 | + | 1.33 | nd | nd | nd | nd | 2 | 2 | 8 |
| kp3 | 11 | + | 1.85 | + | 2.07 | + | 2.01 | 2 | 0.5 | 2 |
| kp4 | 11 | + | 1.31 | nd | nd | nd | nd | 8 | 4 | ＞64 |
| kp5 | 11 | + | 1.98 | + | 2.2 | + | 2.13 | 2 | 0.5 | 32 |
| kp7 | 11 | + | 1.53 | nd | nd | nd | nd | 2 | 2 | 4 |
| kp8 | 11 | + | 1.61 | nd | nd | nd | nd | 2 | 2 | 4 |
| kp9 | 11 | + | 1.74 | nd | nd | nd | nd | ＞128 | 2 | 16 |
| kp10 | 11 | + | 2.99 | nd | nd | nd | nd | ＞128 | ＞128 | ＞64 |
| kp11 | 11 | + | 1.66 | nd | nd | nd | nd | 4 | 2 | 64 |
| kp12 | 11 | + | 1.83 | + | 1.95 | + | 1.88 | 2 | 0.5 | 4 |
| kp14 | 11 | + | 1.41 | nd | nd | nd | nd | 2 | 0.5 | 4 |
| kp15 | 11 | + | 1.75 | + | 1.89 | + | 1.77 | 4 | 0.5 | 4 |
| kp16 | 11 | + | 1.83 | + | 2.03 | + | 1.93 | 2 | 1 | 4 |
| kp17 | 11 | + | 2.98 | nd | nd | nd | nd | 8 | 4 | 32 |
| kp18 | 11 | + | 2.99 | nd | nd | nd | nd | 4 | 4 | 32 |
| kp19 | 11 | + | 1.86 | nd | nd | + | 1.78 | 2 | 0.5 | 8 |
| kp20 | 11 | + | 4.45 | nd | nd | nd | nd | 8 | 2 | ＞64 |
| kp21 | 11 | + | 4.04 | nd | nd | nd | nd | 2 | 0.125 | ≤0.03 |
| kp22 | 11 | + | 1.77 | nd | nd | + | 1.59 | 2 | 1 | 8 |
| kp23 | 11 | + | 2.98 | nd | nd | nd | nd | 4 | 1 | 8 |
| kp24 | 11 | + | 1.67 | nd | nd | nd | nd | 1 | 0.25 | 2 |
| kp26 | 11 | + | 3.15 | nd | nd | nd | nd | 4 | 2 | 64 |
| kp28 | 11 | + | 1.55 | nd | nd | nd | nd | 16 | 1 | 4 |
| kp29 | 11 | + | 1.56 | nd | nd | nd | nd | 2 | 0.5 | 1 |
| kp30 | 11 | + | 1.72 | + | 1.96 | + | 1.73 | 2 | 1 | 2 |
| kp31 | 11 | + | 1.65 | + | 1.84 | + | 1.68 | 2 | 2 | 8 |
| kp32 | 11 | + | 1.86 | + | 2.06 | + | 1.81 | 4 | 0.5 | 4 |
| kp33 | 11 | + | 2.91 | nd | nd | nd | nd | 4 | 2 | 16 |
| kp34 | 11 | + | 4.19 | nd | nd | nd | nd | 8 | 2 | 32 |
| kp35 | 11 | + | 1.82 | nd | nd | + | 1.74 | 2 | 1 | 8 |
| kp37 | 11 | + | 1.88 | nd | nd | + | 1.75 | 4 | 2 | 16 |
| kp38 | 11 | + | 2.84 | nd | nd | nd | nd | 4 | 1 | 8 |
| kp39 | 11 | + | 1.7 | nd | nd | nd | nd | 2 | 0.25 | 0.5 |
| kp40 | 11 | + | 3.13 | nd | nd | nd | nd | 2 | 2 | 8 |
| kp41 | 11 | + | 3.3 | nd | nd | nd | nd | 4 | 4 | 32 |
| kp42 | 11 | + | 4.2 | nd | nd | nd | nd | 8 | 2 | ＞64 |
| kp46 | 11 | + | 2.97 | nd | nd | nd | nd | 8 | 2 | 32 |
| kp51 | 11 | + | 1.77 | + | 1.86 | + | 1.75 | 2 | 0.5 | 8 |
| kp53 | 11 | + | 1.76 | nd | nd | + | 1.71 | 2 | 1 | 4 |
| kp54 | 11 | + | 1.35 | nd | nd | + | 1.32 | 2 | 2 | 8 |
| kp55 | 11 | + | 0.67 | nd | nd | nd | nd | 2 | 0.5 | 2 |
| kp56 | 11 | + | 1.55 | + | 1.84 | + | 1.53 | 2 | 1 | 4 |
| kp59 | 11 | + | 1.65 | nd | nd | nd | nd | 2 | 1 | 4 |
| kp63 | 11 | + | 1.37 | + | 1.82 | + | 1.28 | 4 | 1 | 8 |
| kp66 | 11 | + | 1.23 | + | 1.84 | + | 1.24 | 2 | 2 | 1 |
| kp69 | 11 | + | 0.76 | nd | nd | nd | nd | 4 | 1 | 32 |
| kp70 | 11 | + | 1.08 | + | 1.54 | + | 1.14 | 2 | 0.5 | 4 |
| kp71 | 11 | + | 1.99 | + | 8.32 | + | 2 | 4 | 1 | 8 |
| kp73 | 11 | + | 1.1 | nd | nd | nd | nd | 2 | 0.5 | 8 |
| kp80 | 11 | + | 0.92 | nd | nd | nd | nd | 4 | 1 | 8 |
| kp82 | 11 | + | 1.87 | + | 2.31 | + | 1.95 | 4 | 2 | 8 |
| kp84 | 11 | + | 1.96 | nd | nd | + | 2.18 | 2 | 0.5 | 8 |
| kp86 | 11 | + | 1.96 | + | 2.35 | + | 2.07 | 2 | 1 | 2 |
| kp87 | 11 | + | 2.01 | + | 2.41 | + | 2.21 | 2 | 1 | 2 |
| kp88 | 11 | + | 1.7 | nd | nd | nd | nd | 2 | 0.5 | 1 |
| kp89 | 11 | + | 1.89 | + | 2.34 | + | 2.21 | 4 | 2 | 2 |
| kp91 | 11 | + | 2.11 | nd | nd | nd | nd | 8 | 4 | 64 |
| kp93 | 11 | + | 3.33 | nd | nd | nd | nd | 4 | 2 | 64 |
| kp94 | 11 | + | 1.5 | nd | nd | nd | nd | 2 | 2 | 4 |
| kp97 | 11 | + | 1.86 | nd | nd | + | 1.93 | 2 | 1 | 32 |
| kp100 | 11 | + | 1.94 | nd | nd | + | 2.12 | 1 | 4 | 32 |
| kp103 | 11 | + | 2.83 | nd | nd | nd | nd | 8 | 4 | 32 |
| kp106 | 11 | + | 1.82 | + | 2.3 | + | 2.03 | 4 | 1 | 4 |
| kp111 | 11 | + | 1.56 | + | 2.12 | + | 1.71 | 2 | 1 | 4 |
| kp116 | 11 | + | 1.78 | + | 2.17 | + | 1.9 | 2 | 2 | 8 |
| kp117 | 11 | + | 4.42 | nd | nd | + | 1.9 | 2 | 0.5 | 0.25 |
| kp118 | 11 | + | 1.46 | nd | nd | nd | nd | 2 | 0.5 | 8 |
| kp121 | 11 | + | 1.33 | + | 1.87 | + | 8.44 | 4 | 1 | 8 |
| kp122 | 11 | + | 1.65 | + | 2.02 | + | 1.76 | ＞128 | 1 | 4 |
| kp124 | 11 | + | 1.84 | + | 2.36 | + | 2.17 | 2 | 0.5 | 4 |
| kp125 | 11 | + | 1.24 | nd | nd | nd | nd | 4 | 0.5 | 8 |
| kp126 | 11 | + | 0.58 | nd | nd | nd | nd | 2 | 1 | 2 |
| kp130 | 11 | + | 3.24 | nd | nd | + | 2.02 | ＞128 | ＞128 | ＞64 |
| kp131 | 11 | + | 0.98 | nd | nd | + | 1.2 | 2 | 1 | 2 |
| kp138 | 11 | + | 1.39 | nd | nd | nd | nd | 2 | 1 | 8 |
| kp139 | 11 | + | 0.76 | nd | nd | nd | nd | 4 | 1 | 8 |
| kp142 | 11 | + | 0.98 | nd | nd | + | 1.18 | 0.5 | 128 | ＞64 |
| kp143 | 11 | + | 0.81 | nd | nd | nd | nd | 2 | 2 | 4 |
| kp144 | 11 | + | 0.56 | nd | nd | nd | nd | 0.5 | 0.25 | ≤0.03 |
| kp146 | 11 | + | 2.15 | nd | nd | nd | nd | ＞128 | 1 | 8 |
| kp148 | 11 | + | 2.37 | nd | nd | + | 1.36 | 2 | 0.25 | 0.0625 |
| kp149 | 11 | + | 0.94 | nd | nd | + | 0.98 | ＞128 | 16 | 32 |
| kp151 | 11 | + | 1.67 | nd | nd | + | 1.68 | ＞128 | 1 | 8 |
| kp152 | 11 | + | 1.53 | nd | nd | + | 1.44 | 2 | 0.5 | 8 |
| kp153 | 11 | + | 1.24 | nd | nd | + | 1.15 | 2 | 1 | 16 |
| kp154 | 11 | + | 0.93 | nd | nd | nd | nd | 2 | 0.5 | 4 |
| kp156 | 11 | + | 1.12 | nd | nd | + | 1.24 | 2 | 1 | 2 |
| kp158 | 11 | + | 1.22 | nd | nd | nd | nd | 1 | 0.25 | ≤0.03 |
| kp159 | 11 | + | 1.46 | + | 1.95 | nd | nd | ＞128 | 2 | 32 |
| kp160 | 11 | + | 0.74 | + | 1.34 | + | 0.92 | 2 | 0.5 | 32 |
| kp161 | 11 | + | 1.13 | + | 1.67 | + | 2.31 | 4 | 0.5 | 2 |
| kp162 | 11 | + | 3.32 | nd | nd | nd | nd | 4 | 2 | 16 |
| kp164 | 11 | + | 1.21 | nd | nd | nd | nd | 2 | 0.5 | 4 |
| kp165 | 11 | + | 2.93 | nd | nd | nd | nd | 2 | 1 | 64 |
| kp166 | 11 | + | 1.94 | + | 2.19 | + | 3.92 | 2 | 1 | 2 |
| kp167 | 11 | + | 1.59 | nd | nd | nd | nd | 2 | 2 | 2 |
| kp168 | 11 | + | 2.85 | nd | nd | nd | nd | 4 | 2 | 64 |
| kp169 | 11 | + | 0.91 | + | 10.13 | + | 2.23 | 16 | 0.5 | 4 |
| kp171 | 11 | + | 1.56 | nd | nd | nd | nd | 2 | 1 | 1 |
| kp173 | 11 | + | 3.2 | nd | nd | nd | nd | 4 | 2 | 16 |
| kp174 | 11 | + | 1.68 | + | 2.1 | + | 1.99 | 4 | 0.5 | 2 |
| kp175 | 11 | + | 2.56 | nd | nd | nd | nd | 4 | 2 | 4 |
| kp176 | 11 | + | 1.76 | + | 2.31 | + | 2.08 | 2 | 2 | 2 |
| kp177 | 11 | + | 1 | + | 18.7 | + | 2.22 | 16 | 0.5 | 4 |
| kp178 | 11 | + | 2.1 | + | 2.44 | + | 2.1 | 2 | 0.5 | 1 |
| kp179 | 11 | + | 1.59 | nd | nd | nd | nd | 2 | 0.5 | 1 |
| kp180 | 11 | + | 1.66 | nd | nd | nd | nd | 1 | 0.5 | 1 |
| kp181 | 11 | + | 3.11 | nd | nd | nd | nd | 4 | 1 | 64 |
| kp182 | 11 | + | 1.57 | nd | nd | nd | nd | 2 | 0.5 | 4 |
| kp183 | 11 | + | 1.22 | + | 2.12 | + | 2.04 | 16 | 1 | 8 |
| kp184 | 11 | + | 7.38 | nd | nd | nd | nd | 8 | 4 | ＞64 |
| kp185 | 11 | + | 1.78 | + | 2.2 | + | 2.03 | 2 | 0.5 | 1 |
| kp186 | 11 | + | 0.96 | + | 1.33 | + | 1.74 | 2 | 0.5 | 2 |
| kp187 | 11 | + | 1.79 | + | 2.13 | + | 1.92 | 2 | 0.5 | 2 |
| kp188 | 11 | + | 1.97 | + | 2.45 | + | 4.2 | 4 | 1 | 2 |
| kp189 | 11 | + | 1.95 | + | 2.53 | + | 2.28 | 2 | 0.5 | 2 |
| kp190 | 11 | + | 1.81 | + | 2.22 | + | 2 | 2 | 0.5 | 4 |
| kp192 | 11 | + | 1.78 | + | 2.21 | + | 1.99 | 2 | 0.5 | 2 |
| kp193 | 11 | + | 2.05 | nd | nd | nd | nd | 2 | 0.5 | 4 |
| kp194 | 11 | + | 2.26 | + | 2.57 | + | 4.26 | 2 | 0.5 | 1 |
| kp195 | 11 | + | 4.84 | nd | nd | nd | nd | 4 | 2 | 32 |
| kp196 | 11 | + | 2.27 | + | 2.61 | + | 4.14 | 4 | 1 | 4 |
| kp197 | 11 | + | 2.34 | + | 2.75 | + | 2.31 | 2 | 0.5 | 4 |
| kp198 | 11 | + | 1.94 | nd | nd | nd | nd | 2 | 0.5 | 2 |
| kp199 | 11 | + | 5.75 | nd | nd | nd | nd | 8 | 4 | ＞64 |
| kp200 | 11 | + | 4.9 | nd | nd | nd | nd | 8 | 2 | 16 |
| kp201 | 11 | + | 2.3 | nd | nd | nd | nd | 2 | 1 | 2 |
| kp202 | 11 | + | 4.58 | nd | nd | nd | nd | 4 | 2 | 64 |
| kp203 | 11 | + | 2.31 | + | 2.69 | + | 6.42 | 2 | 1 | 2 |
| kp204 | 11 | + | 2.24 | + | 2.63 | + | 3.97 | 4 | 2 | 8 |
| kp207 | 11 | + | 2.4 | + | 2.86 | + | 4.41 | 2 | 0.5 | 2 |
| kp208 | 11 | + | 3.91 | nd | nd | nd | nd | 4 | 1 | 64 |
| kp209 | 11 | + | 1.07 | + | 15.49 | + | 2.05 | 8 | 0.5 | 4 |
| kp211 | 11 | + | 2.13 | nd | nd | nd | nd | 4 | 0.5 | 2 |
| kp212 | 11 | + | 3.61 | nd | nd | nd | nd | 4 | 1 | 32 |
| kp213 | 11 | + | 1.43 | nd | nd | nd | nd | 2 | 0.5 | 4 |
| kp214 | 11 | + | 0.59 | nd | nd | nd | nd | 2 | 0.5 | 2 |
| kp215 | 11 | + | 1.31 | nd | nd | nd | nd | 2 | 0.25 | 0.5 |
| kp222 | 11 | + | 0.44 | nd | nd | nd | nd | 4 | 0.5 | 32 |
| kp226 | 11 | + | 1.54 | + | 1.71 | + | 2.83 | 2 | 0.5 | 2 |
| kp227 | 11 | + | 2.61 | nd | nd | nd | nd | 8 | 4 | ＞64 |
| kp228 | 11 | + | 1.25 | + | 1.46 | + | 1.28 | 2 | 1 | 4 |
| kp231 | 11 | + | 1.84 | nd | nd | nd | nd | 8 | 4 | ＞64 |
| kp232 | 11 | + | 1.77 | nd | nd | nd | nd | 4 | 1 | 32 |
| kp236 | 11 | + | 3.6 | nd | nd | nd | nd | 2 | 0.5 | 8 |
| kp237 | 11 | + | 1 | nd | nd | nd | nd | 4 | 2 | 64 |
| kp239 | 11 | + | 1.22 | + | 1.56 | + | 1.4 | 2 | 0.5 | 2 |
| kp241 | 11 | + | 1.5 | nd | nd | nd | nd | 128 | 32 | 64 |
| kp242 | 11 | + | 0.61 | nd | nd | nd | nd | 4 | 0.5 | 4 |
| kp245 | 11 | + | 0.74 | + | 6.14 | + | 1.43 | 4 | 0.25 | 4 |
| kp246 | 11 | + | 0.73 | nd | nd | nd | nd | 2 | 1 | 4 |
| kp248 | 11 | + | 2.28 | + | 2.38 | + | 2.15 | 8 | 4 | 64 |
| kp250 | 11 | + | 2.31 | nd | nd | nd | nd | 1 | 0.5 | ≤0.03 |
| kp252 | 11 | + | 0.73 | nd | nd | nd | nd | 2 | 0.25 | 2 |
| kp253 | 11 | + | 1.14 | nd | nd | nd | nd | 2 | 2 | 0.25 |
| kp255 | 11 | + | 0.76 | nd | nd | nd | nd | 2 | 0.5 | 4 |
| kp256 | 11 | + | 0.85 | nd | nd | nd | nd | 2 | 0.5 | 4 |
| kp258 | 11 | + | 1.91 | + | 2.06 | + | 1.88 | 8 | 4 | ＞64 |
| kp259 | 11 | + | 2.43 | nd | nd | nd | nd | 2 | 0.5 | 4 |
| kp262 | 11 | + | 2.94 | nd | nd | nd | nd | 2 | 1 | 2 |
| kp263 | 11 | + | 2.35 | nd | nd | nd | nd | 2 | 2 | 2 |
| kp264 | 11 | + | 0.96 | nd | nd | nd | nd | 2 | 0.5 | 4 |
| kp266 | 11 | + | 0.95 | nd | nd | nd | nd | 2 | 2 | 1 |
| kp267 | 11 | + | 0.88 | nd | nd | nd | nd | 1 | 2 | 0.5 |
| kp268 | 11 | + | 2.02 | + | 15.3 | + | 2.23 | 8 | 2 | 2 |
| kp270 | 11 | + | 3.8 | nd | nd | nd | nd | ＞128 | 0.125 | 0.125 |
| kp273 | 11 | + | 0.98 | nd | nd | nd | nd | 1 | 2 | 1 |
| kp276 | 11 | + | 1.22 | + | 1.7 | + | 1.41 | 2 | 2 | 1 |
| kp278 | 11 | + | 1 | nd | nd | nd | nd | 1 | 2 | 2 |
| kp283 | 11 | + | 1.54 | nd | nd | + | 1.6 | 1 | 0.5 | 0.25 |
| kp284 | 11 | + | 0.72 | nd | nd | nd | nd | 2 | 2 | 8 |
| kp286 | 11 | + | 1.69 | + | 2.44 | + | 1.87 | 2 | 4 | 0.5 |
| kp287 | 11 | + | 0.95 | + | 1.94 | + | 1.34 | 2 | 2 | 1 |
| kp288 | 11 | + | 0.99 | nd | nd | nd | nd | 2 | 2 | 2 |
| kp289 | 11 | + | 3.04 | nd | nd | nd | nd | 4 | 4 | 32 |
| kp290 | 11 | + | 1.02 | nd | nd | nd | nd | 2 | 2 | 2 |
| KP293 | 11 | + | 1.26 | + | 2.02 | + | 1.37 | 2 | 1 | 0.5 |
| KP294 | 11 | + | 1.17 | + | 1.88 | + | 1.36 | 2 | 1 | 0.5 |
| KP295 | 11 | + | 1.93 | + | 2.34 | + | 1.86 | 2 | 2 | 0.5 |
| KP298 | 11 | + | 1.25 | + | 2.05 | + | 1.59 | 2 | 1 | 1 |
| KP299 | 11 | + | 1.16 | + | 2.26 | + | 1.62 | 128 | 64 | ＞64 |
| KP300 | 11 | + | 1.72 | nd | nd | + | 1.54 | 1 | 0.25 | 0.125 |
| KP302 | 11 | + | 0.93 | + | 1.81 | + | 1.41 | 128 | 2 | 8 |
| KP303 | 11 | + | 1.11 | + | 1.79 | + | 1.13 | 2 | 2 | 1 |
| KP305 | 11 | + | 3.28 | nd | nd | nd | nd | 2 | 2 | 0.5 |
| KP312 | 11 | + | 3.35 | nd | nd | nd | nd | 2 | 1 | 0.0625 |
| KP313 | 11 | + | 1.28 | nd | nd | + | 1.25 | 2 | 2 | 0.25 |
| KP316 | 11 | + | 2.99 | nd | nd | nd | nd | 4 | 2 | 1 |
| KP318 | 11 | + | 1.28 | nd | nd | + | 1.28 | 2 | 0.5 | 0.25 |
| KP319 | 11 | + | 0.75 | + | 9.15 | + | 1.18 | 8 | 1 | 2 |
| KP320 | 11 | + | 1.06 | + | 1.88 | + | 1.18 | 4 | 2 | 1 |
| KP325 | 11 | + | 2.5 | + | 3.47 | + | 2.63 | 2 | 0.5 | 0.5 |
| KP335 | 11 | + | 0.94 | + | 1.83 | + | 1.2 | 4 | 1 | 2 |
| KP337 | 11 | + | 0.93 | + | 1.7 | + | 1.04 | 4 | 1 | 1 |
| KP344 | 11 | + | 2.11 | + | 2.38 | + | 2.2 | 2 | 2 | 0.5 |
| KP345 | 11 | + | 1.35 | + | 1.92 | + | 1.36 | 4 | 4 | 4 |
| KP347 | 11 | + | 1.44 | nd | nd | + | 1.4 | 0.5 | 0.25 | 0.125 |
| KP348 | 11 | + | 1.41 | nd | nd | + | 1.39 | 2 | 0.25 | 2 |
| KP350 | 11 | + | 2.12 | + | 2.54 | + | 1.99 | 5 | 4 | 64 |
| KP351 | 11 | + | 0.94 | + | 1.21 | + | 0.87 | 4 | 4 | 8 |
| KP353 | 11 | + | 3.18 | nd | nd | nd | nd | 1 | 0.5 | 0.0625 |
| KP354 | 11 | + | 1.12 | + | 2 | + | 1.54 | 4 | 1 | 8 |
| KP355 | 11 | + | 1.17 | + | 8.61 | + | 1.79 | 8 | 0.25 | 4 |
| KP357 | 11 | + | 1.02 | + | 1.92 | + | 1.35 | 4 | 0.5 | 4 |
| KP364 | 11 | + | 1.3 | + | 13.45 | + | 2.5 | 8 | 0.5 | 4 |
| KP366 | 11 | + | 0.82 | + | 5.5 | + | 1.07 | 8 | 0.5 | 4 |
| KP368 | 11 | + | 1.25 | + | 13.51 | + | 2.1 | 16 | 0.25 | 4 |
| KP372 | 11 | + | 1.74 | nd | nd | nd | nd | 1 | 0.5 | ≤0.03 |
| kp373 | 11 | + | 1.48 | + | 2.41 | + | 1.71 | 2 | 1 | 8 |
| KP374 | 11 | + | 0.84 | + | 5.73 | + | 1.17 | 8 | 1 | 4 |
| KP376 | 11 | + | 1.14 | + | 1.75 | + | 1.18 | 2 | 1 | 2 |
| KP377 | 11 | + | 0.92 | + | 3.56 | + | 1.23 | 8 | 0.5 | 4 |
| kp378 | 11 | + | 1.67 | + | 2.21 | + | 1.76 | 2 | 1 | 4 |
| KP379 | 11 | + | 0.79 | + | 3.85 | + | 0.94 | 16 | 0.5 | 8 |
| KP380 | 11 | + | 0.85 | + | 3.58 | + | 1.12 | 16 | 0.5 | 4 |
| KP385 | 11 | + | 1.1 | + | 7.85 | + | 1.45 | 8 | 0.5 | 4 |
| KP393 | 11 | + | 2.5 | nd | nd | nd | nd | 1 | 0.5 | ≤0.03 |
| KP394 | 11 | + | 1.26 | nd | nd | + | 1.16 | 2 | 0.5 | 4 |
| KP397 | 11 | + | 1.43 | + | 2 | + | 1.49 | 4 | 1 | 4 |
| KP411 | 11 | + | 1.87 | + | 2.31 | + | 1.85 | 2 | 2 | 1 |
| KP413 | 11 | + | 0.96 | nd | nd | + | 1 | 2 | 4 | 32 |
| KP421 | 11 | + | 1.4 | nd | nd | + | 1.51 | 2 | 1 | 1 |
| KP424 | 11 | + | 1.31 | + | 1.62 | + | 1.28 | 2 | 1 | 4 |
| KP426 | 11 | + | 0.87 | nd | nd | nd | nd | 4 | 2 | 2 |
| KP428 | 11 | + | 1.26 | + | 1.71 | + | 1.35 | 2 | 1 | 2 |
| KP430 | 11 | + | 1.16 | nd | nd | + | 0.94 | 4 | 2 | 1 |
| KP431 | 11 | + | 0.77 | nd | nd | nd | nd | 1 | 1 | 1 |
| KP434 | 11 | + | 1.62 | + | 2.04 | + | 1.69 | 2 | 1 | 2 |
| KP436 | 11 | + | 2.42 | + | 3.64 | + | 2.39 | 2 | 1 | 4 |
| KP438 | 11 | + | 2.35 | + | 2.87 | + | 2.24 | 2 | 1 | 1 |
| KP439 | 11 | + | 1.11 | + | 1.73 | nd | nd | 2 | 0.5 | 2 |
| KP442 | 11 | + | 1.54 | nd | nd | nd | nd | 4 | 1 | 4 |
| KP443 | 11 | + | 1.23 | nd | nd | nd | nd | 2 | 0.5 | 4 |
| KP445 | 11 | + | 1.73 | + | 2.23 | + | 1.65 | 2 | 1 | 8 |
| KP446 | 11 | + | 1.51 | nd | nd | nd | nd | 2 | 1 | 4 |
| KP447 | 11 | + | 1.26 | nd | nd | nd | nd | 2 | 2 | 8 |

+, positive;nd,not detected

Table S2. Antimicrobial susceptibilities of CRKP isolates and transconjugants in our study.

|  | CAZ | PTZ | MEM | IMI | ETP | CZA | MEV | I/R |
| --- | --- | --- | --- | --- | --- | --- | --- | --- |
| KCt_E1 | **128** | **＞512** | **128** | **64** | **>128** | 2 | 2 | 2 |
| KNs_E1 | **＞128** | **＞512** | **＞128** | **128** | **>128** | 4 | **64** | 2 |
| KB16_E2 | **＞128** | **＞512** | **64** | **16** | **>128** | **16** | 4 | 0.5 |
| KB21_P1 | **64** | **＞512** | **128** | **32** | **>128** | 2 | 1 | 1 |
| KB20_P2 | **128** | **＞512** | **＞128** | **64** | **>128** | 4 | **16** | 2 |
| KB25_E1 | **＞128** | **＞512** | **128** | **32** | **>128** | 4 | 2 | 0.5 |
| KB20_E1 | **128** | **＞512** | **＞128** | **64** | **>128** | 4 | 4 | 2 |
| KB19_E1 | **＞128** | **＞512** | **128** | **32** | **>128** | 2 | 2 | 2 |
| KB16_E3 | **＞128** | **＞512** | **128** | **32** | **>128** | **16** | 4 | 0.5 |
| KB7_E | **＞128** | **＞512** | **128** | **32** | **>128** | 2 | 1 | 0.5 |
| KB10_E | **64** | **＞512** | **128** | **32** | **>128** | 2 | 1 | 0.5 |
| KB9_E | **64** | **＞512** | **128** | **32** | **>128** | 1 | 1 | 0.5 |
| KB18_E | **128** | **＞512** | **＞128** | **64** | **>128** | 4 | **64** | 1 |
| KB22_E1 | **128** | **＞512** | **＞128** | **64** | **>128** | 2 | 4 | 0.5 |
| KB16_P2 | **＞128** | **＞512** | **＞128** | **32** | **>128** | **16** | 8 | 1 |
| KB20_P3 | **＞128** | **＞512** | **＞128** | **128** | **>128** | 8 | **＞64** | **4** |
| KB25_P1 | **＞128** | **＞512** | **64** | **32** | **>128** | 2 | 1 | 0.5 |
| KB10_P1 | **＞128** | **＞512** | **128** | **32** | **>128** | 2 | 2 | 0.5 |
| KB25_P2 | **＞128** | **＞512** | **64** | **32** | **>128** | 2 | 2 | 0.5 |
| KB10_P2 | **＞128** | **＞512** | **128** | **64** | **>128** | 4 | 2 | 1 |
| KB16_P1 | **＞128** | **＞512** | **＞128** | **64** | **>128** | 2 | 2 | 0.5 |
| KB25_P3 | **＞128** | **＞512** | **128** | **32** | **>128** | 2 | 4 | 0.5 |
| KCt_E2 | **128** | **＞512** | **＞128** | **64** | **>128** | 4 | **32** | 2 |
| KNs_E2 | **＞128** | **＞512** | **128** | **64** | **>128** | 4 | 4 | 1 |
| KB16_E1 | **＞128** | **＞512** | **128** | **32** | **>128** | 2 | 4 | 0.5 |
| KB1_P | **128** | **＞512** | **128** | **32** | **>128** | 2 | 2 | 0.5 |
| KB21_P2 | **＞128** | **＞512** | **＞128** | **128** | **>128** | 8 | **＞64** | **4** |
| KB19_P | **＞128** | **＞512** | **＞128** | **64** | **>128** | 8 | **16** | 2 |
| KB20_P1 | **64** | **＞512** | **128** | **32** | **>128** | 2 | 2 | 1 |
| KB25_E2 | **＞128** | **＞512** | **＞128** | **64** | **>128** | 4 | **64** | 2 |
| KB20_E2 | **＞128** | **＞512** | **128** | **32** | **>128** | 2 | 2 | 1 |
| KB19_E2 | **＞128** | **＞512** | **＞128** | **64** | **>128** | 4 | 8 | 2 |
| J53 | 0.25 | 2 | 0.03 | 0.25 | 0.004 | 0.25 | ＜0.03 | 0.25 |
| J53-pKB16_E2_KPC | **128** | **64** | **4** | 2 | **2** | 1 | ＜0.03 | 0.25 |
| J53-pKB16_E2_SHV | **128** | 32 | 0.03 | 0.25 | 0.06 | 1 | ＜0.03 | 0.25 |

CAZ, Ceftazidime; PTZ, Piperacillin/Tazobactam; MEM , Meropenem; IMI, Imipenem; ETP, Ertapenem; CZA, Ceftazidime/avibactam;MEV, Meropenem/Vaborbactam; I/R, Imipenem/Relebactam
